# Prevalence and factors associated with Chinese herbal medicine use among middle-aged and older Chinese adults with diabetes mellitus

**DOI:** 10.3389/fphar.2025.1482228

**Published:** 2025-05-22

**Authors:** Yanting Chen, Leying Lin, Shanwei Sun, Kaiwang Cui, Qingrong Wu

**Affiliations:** ^1^ Rheumatology Department, The First Affiliated Hospital of Guangdong Pharmaceutical University, Guangzhou, Guangdong, China; ^2^ School of Basic Medicine, Gannan Medical University, Ganzhou, Jiangxi, China; ^3^ Department of Respiratory and Critical Care Medicine, Ganzhou Key Laboratory of Respiratory Diseases, Ganzhou Institute of Respiratory Diseases, Ganzhou Fifth People’s Hospital, Ganzhou, Jiangxi, China; ^4^ Department of Pharmacy, Ganzhou Fifth People’s Hospital, Ganzhou, Jiangxi, China

**Keywords:** complementary and alternative medicine, Chinese herbal medicine, diabetes, prevalence, associated factors

## Abstract

**Background:**

The effectiveness of traditional Chinese medicine (TCM) in treating diabetes has been confirmed in China and globally. However, research on the use of Chinese herbal medicine (CHM) among middle-aged and elderly patients with diabetes and its associated factors is limited. This study aims to explore the prevalence of CHM use among these patients and its associated factors.

**Methods:**

This study utilized data from the China Health and Retirement Longitudinal Study, which covers Chinese adults aged ≥45 years. A cross-sectional analysis was conducted on 3,347 participants who used CHM for diabetes treatment. Multivariate logistic regression models were employed to identify key factors (including demographic characteristics, health status, and healthcare utilization) that predict CHM use among patients with diabetes.

**Results:**

The prevalence of CHM use for diabetes was 10.8% among middle-aged and older Chinese patients with diabetes. Compared to patients with diabetes who did not use CHM, those who did were more likely to be older (OR = 1.31; 95% CI = 1.04, 1.65), visit TCM hospitals (OR = 1.24; 95% CI = 1.01, 1.53), engage in self-treatment (OR = 1.90; 95% CI = 1.38, 2.61), have kidney disease (OR = 1.50; 95% CI = 1.05, 2.14), and have asthma (OR = 2.19; 95% CI = 1.29, 3.70). In the combined effect analysis, patients with both kidney disease and asthma were most likely to use CHM (OR = 4.20; 95% CI = 1.93, 9.14).

**Conclusion:**

The prevalence of CHM use among middle-aged and elderly Chinese patients with diabetes was relatively low, and was associated with specific health conditions and healthcare behaviors.

## Introduction

Diabetes mellitus is a group of complex metabolic disorders characterized by chronic hyperglycemia, which is characterized by glucose imbalance, insulin resistance, and dysfunction of pancreatic β-cells. Clinically, it presents with symptoms such as polyphagia, polydipsia, polyuria, irritability, weight loss, and obesity (Kretchy et al., 2016). According to the International Diabetes Federation (IDF), approximately 537 million people worldwide have diabetes, with projections suggesting a continued increase in the coming decades (Sun et al., 2022). China currently has the highest number of diabetic patients, with 125 million affected individuals, and this number is expected to double by 2040 (Chinese Diabetes Society and National Office for Primary Diabetes CareNational Office for Primary Diabetes Care, 2022). Consequently, strategies for the prevention and control of diabetes have become an urgent public health priority in China and globally. Traditional treatments for diabetes include lifestyle modifications (Schellenberg et al., 2013; Li et al., 2003), oral hypoglycemic agents (such as metformin, α-glucosidase inhibitors, and insulin sensitizers) (Tran et al., 2015a), and injectable medications (such as insulin and glucagon-like peptide-1) (Tran et al., 2015b). However, despite intensified glycemic control, most diabetic patients experience various complications, and the use of hypoglycemic drugs often results in adverse effects. A meta-analysis has shown that the use of metformin, sulfonylureas, and thiazolidinediones increases the risk of cardiovascular events and mortality in diabetic patients (Rao et al., 2008; Roumie et al., 2012; Lincoff et al., 2007). Long-term use of thiazolidinediones also increases the risks of fractures, lower respiratory infections, and bladder cancer (Loke et al., 2009; Singh et al., 2011; Turner et al., 2014). Therefore, the search for complementary and alternative medicine to prevent or delay the complications and progression of diabetes is a current research focus (Ching et al., 2013; Naja et al., 2014).

Chinese herbal medicine (CHM), an important component of traditional medicine, offers possibilities for achieving this goal. In fact, some general populations and chronic disease patients worldwide rely on traditional medicine to meet their basic healthcare needs, particularly in developing countries, where approximately 70%–95% of the population uses CHM (Pearson et al., 2018; Kifle et al., 2021; Ghorat et al., 2024; El-Nimr et al., 2015; Amaeze et al., 2018). Chronic disease patients often employ CHM to treat and delay the progression of their conditions (Ching et al., 2013; Yildirim and Marakoglu, 2018; Mbizo et al., 2018), and they generally perceive these remedies as natural, safe, and free of adverse side effects.

In China, CHM has been used in the clinical treatment of diabetes for thousands of years. Traditional Chinese medicine (TCM) refers to diabetes as “Xiao Ke” (Guo et al., 2014), which is characterized by symptoms and signs such as sweet urine, dry mouth, thirst, excessive drinking, overeating, emaciation, and fatigue (Ma et al., 2004; Wang et al., 2020). Previous studies have also reported the effectiveness and safety of CHM. CHM has been shown to effectively reduce blood glucose and HbA1c levels in diabetic patients (Bai et al., 2019; Pang et al., 2019), alleviate inflammation (Gui et al., 2016; Sun et al., 2021), and decrease the incidence of complications and mortality caused by elevated blood sugar levels (Huang et al., 2020; Li et al., 2012), thereby improving the disease condition and enhancing the quality of life. The underlying mechanism of blood sugar regulation by CHM involves correction of the instability of the internal environment of the human body caused by pathogenic factors, allowing the body to return to a stable state. The mechanism may involve multiple pathways and targets, such as inhibiting the apoptosis of pancreatic beta cells and regulating insulin microcirculation (Pang et al., 2019; Leng et al., 2020; Xu et al., 2018). In summary, CHM can achieve both symptomatic treatment and correction of the underlying cause of this common yet complex metabolic disease. Additionally, the safety and lack of side effects of CHM reduce the reliance on Western medications and significantly increase their enthusiasm for use.

In China, the use of CHM is not only widespread but also deeply rooted in cultural awareness. A previous study among Chinese diabetic patients has indicated that TCM therapies (Ji et al., 2024; Tian et al., 2016), particularly herbal treatments, play a significant role in diabetes management. However, literature on the prevalence and relevance of herbal use among middle-aged and elderly diabetic patients in China remains scarce. Therefore, this study aims to utilize the China Health and Retirement Longitudinal Study to investigate the prevalence, characteristics, and associated factors of CHM use among middle-aged and elderly diabetic patients in China. Our findings will provide data for healthcare professionals to enhance research on the use and safety of herbal treatments.

## Materials and methods

### Study design and population

This study constitutes a secondary analysis of a nationwide longitudinal survey, namely, the China Health and Retirement Longitudinal Study (CHARLS). CHARLS is an ongoing nationally representative cohort, targeting individuals aged 45 years and above, covering 150 regions and 450 villages/urban communities, employing a stratified multistage probability proportional random sampling method. Detailed information on the cohort design has been reported in previous studies (Zhao et al., 2014). To date, five datasets have been released in 2011, 2013, 2015, 2018, and 2020. The original CHARLS received approval from by the Ethical Review Committee of Peking University (IRB00001052-11015). All procedures adhered to pertinent guidelines and regulations. Furthermore, all participants provided informed consent.

This study extracted data from five surveys published in the CHARLS database on patients with diabetes who used CHM treatment. If a patient had multiple follow-up records, the most recent data were selected. For this study, a total of 3,347 participants aged 45 years or older who used CHM for diabetes treatment were included.

### CHM use

Participants who responded “yes” to the following question were considered CHM users: “Are you currently taking CHM to treat diabetes or its complications?”

### Demographic data

Demographic data were gathered using a standardized questionnaire that encompassed variables such as age, gender, educational attainment, place of residence, marital status, financial stability, smoking habits, and alcohol use. Age was stratified into two groups: under 65 years and 65 years or older. Educational levels were categorized as illiterate, primary education or less, and secondary education or higher. Residence was dichotomized into urban and rural areas. Marital status was grouped into married and other categories which included unmarried, separated, divorced, and widowed individuals. Financial status was assessed based on whether respondents had received a wage and bonus income in the preceding year, with responses categorized as “yes” or “no.” Smoking and alcohol consumption were classified into three categories: “never,” “former,” and “current.”

### Health services

Health service data included aspects such as insurance status, type of medical facility utilized, and self-treatment practices. The insurance status was determined by querying participants about their current health insurance coverage. A response indicating no insurance was classified as “no,” while any other response was classified as “yes.”

The type of medical facility was determined by asking participants about the medical facilities they visited within the past month. The responses were categorized as “general hospitals,” “specialized hospitals,” and “TCM hospitals.” Self-treatment was evaluated by inquiring about the self-treatment methods used by participants within the past month. A response indicating no self-treatment was categorized as “no,” while any other response was categorized as “yes”.

### Health status

Health status information included variables related to general health, chronic conditions, depression, and life satisfaction. General health was assessed based on participants’ self-reported ratings of their overall health as “good,” “fair,” and “poor.” Chronic conditions were determined by asking participants whether a physician had diagnosed them with hypertension, dyslipidemia, diabetes, cancer, chronic lung disease, liver disease, heart disease, stroke, kidney disease, stomach disease, psychiatric disorders, memory-related diseases, arthritis, or asthma. Depression was assessed using the Center for Epidemiologic Studies Depression Scale, with scores above 12 indicating depression. Life satisfaction was evaluated by asking participants to rate their overall satisfaction with life.

### Statistical analysis

Differences between diabetic patients who used and did not use CHM were analyzed using Chi-square tests. A stepwise backward logistic regression model was used to identify statistically significant factors associated with CHM use among diabetic patients. Variables with P-value <0.2 in the univariate analysis were included in the model. Additionally, subgroup analyses were performed based on common demographic variables, such as age, gender, and residence region. The additive interaction between kidney disease and asthma on CHM use for diabetes treatment was evaluated. Furthermore, the relative excess risk due to interaction (RERI), attributable proportion (AP), and synergy index (S) were calculated. All analyses were performed using Stata 15.0 SE, and a two-sided P-value <0.05 was considered statistically significant.

## Results

### Demographic characteristics

This study included 3,347 diabetic patients, of whom 361 (10.8%) were treated with CHM. No significant differences were observed between diabetic patients who did and did not use CHM in terms of demographic characteristics (age, gender, education, residence, marital status, receive status, and smoking and alcohol consumption) (Table 1).

**TABLE 1:** Associations between CHM use and demographic characteristics in Chinese middle-aged and older adults with diabetes.

Demographic characteristics	CHM use			P
	Total (n = 3,347)	No (n = 2,986)	Yes (n = 361)	
Age				0.052
Middle-aged adults	1,715 (51.24)	1,550 (51.91)	165 (45.71)	
Older adults	1,581 (47.24)	1,389 (46.52)	192 (53.19)	
Gender				0.170
Male	1,451 (43.35)	1,278 (42.80)	173 (47.92)	
Female	1,872 (55.93)	1,686 (56.46)	186 (51.52)	
Education level				0.650
Illiterate	703 (21.00)	634 (21.23)	69 (19.11)	
Primary school and below	1,156 (34.54)	1,028 (34.43)	128 (35.46)	
Middle school and above	1,488 (44.46)	1,324 (44.34)	164 (45.43)	
Area of residence				0.450
Urban	899 (26.86)	807 (27.03)	92 (25.48)	
Village	2,366 (70.69)	2,103 (70.43)	263 (72.85)	
Marital status				0.770
Married	2,598 (77.62)	2,320 (77.70)	278 (77.01)	
Other	749 (22.38)	666 (22.30)	83 (22.99)	
Income				0.210
Yes	574 (17.15)	524 (17.55)	50 (13.85)	
No	2,646 (79.06)	2,349 (78.67)	297 (82.27)	
Smoking status				0.400
Never	2,051 (61.28)	1,828 (61.22)	223 (61.77)	
Ever smoker	548 (16.37)	499 (16.71)	49 (13.57)	
Current smoker	672 (20.08)	592 (19.83)	80 (22.16)	
Alcohol consumption				0.380
Never drinker	2,143 (64.03)	1,915 (64.13)	228 (63.16)	
Former drinker	282 (8.43)	244 (8.17)	38 (10.53)	
Current drinker	916 (27.37)	821 (27.49)	95 (26.32)	

Data are presented as number (proportion, %).

Missing data: Age = 51, Gender = 24, Residential address = 82, Income = 127, Smoking status = 76, Alcohol consumption = 6.

### Health services

Regarding health services, diabetic patients who used CHM had higher rates of visiting Chinese medicine hospitals and engaging in self-treatment compared to those who did not use CHM. However, there were no significant differences in insurance status or proportion of visits to general hospitals or specialized hospitals between the two groups (Table 2).

**TABLE 2:** Associations between CHM use and health services in Chinese middle-aged and older adults with diabetes.

Health services	CHM use			P
	Total (n = 3,347)	No (n = 2,986)	Yes (n = 361)	
Insurance status				0.310
Yes	2,905 (86.79)	2,598 (87.01)	307 (85.04)	
No	395 (11.80)	349 (11.69)	46 (12.74)	
General hospital				0.800
No	2,852 (85.21)	2,546 (85.26)	306 (84.76)	
Yes	495 (14.79)	440 (14.74)	55 (15.24)	
Specialized hospital				0.340
No	3,285 (98.15)	2,933 (98.23)	352 (97.51)	
Yes	62 (1.85)	53 (1.77)	9 (2.49)	
Chinese Medicine hospital				0.030
No	3,271 (97.73)	2,924 (97.92)	347 (96.12)	
Yes	76 (2.27)	62 (2.08)	14 (3.88)	
Self-treatment				<0.001
No	821 (24.53)	766 (25.65)	55 (15.24)	
Yes	2,508 (74.93)	2,204 (73.81)	304 (84.21)	

Data are presented as number (proportion, %).

Missing data: Insurance status = 47, Self-treatment = 18.

### Health status

As for health status, diabetic patients treated with CHM were more likely to rate their health status as poor and have stroke, kidney disease, and asthma compared to those not treated with CHM. No significant differences were observed between the two groups in terms of other health status variables, including other chronic diseases, depression, and satisfaction (Table 3).

**TABLE 3:** Associations between CHM use and health status in Chinese middle-aged and older adults with diabetes.

Health status	CHM use			P
	Total (n = 3,347)	No (n = 2,986)	Yes (n = 361)	
General health status				0.041
Good	288 (8.60)	266 (8.91)	22 (6.09)	
Fair	1,875 (56.02)	1,682 (56.33)	193 (53.46)	
Poor	1,184 (35.37)	1,038 (34.76)	146 (40.44)	
Hypertension				0.480
No	2,555 (76.34)	2,274 (76.16)	281 (77.84)	
Yes	792 (23.66)	712 (23.84)	80 (22.16)	
Dyslipidemia				0.620
No	2,584 (77.20)	2,309 (77.33)	275 (76.18)	
Yes	763 (22.80)	677 (22.67)	86 (23.82)	
Cancer				0.440
No	3,273 (97.79)	2,922 (97.86)	351 (97.23)	
Yes	74 (2.21)	64 (2.14)	10 (2.77)	
Chronic lung diseases				0.300
No	3,044 (90.95)	2,721 (91.13)	323 (89.47)	
Yes	303 (9.05)	265 (8.87)	38 (10.53)	
Liver disease				0.130
No	3,171 (94.74)	2,835 (94.94)	336 (93.07)	
Yes	176 (5.26)	151 (5.06)	25 (6.93)	
Heart attack				0.071
No	2,851 (85.18)	2,555 (85.57)	296 (81.99)	
Yes	496 (14.82)	431 (14.43)	65 (18.01)	
Stroke				0.025
No	3,054 (91.25)	2,736 (91.63)	318 (88.09)	
Yes	293 (8.75)	250 (8.37)	43 (11.91)	
Kidney disease				0.002
No	3,058 (91.37)	2,744 (91.90)	314 (86.98)	
Yes	289 (8.63)	242 (8.10)	47 (13.02)	
Stomach disease				0.300
No	2,931 (87.57)	2,621 (87.78)	310 (85.87)	
Yes	416 (12.43)	365 (12.22)	51 (14.13)	
Emotional problems				0.460
No	3,264 (97.52)	2,914 (97.59)	350 (96.95)	
Yes	83 (2.48)	72 (2.41)	11 (3.05)	
Memory-related disease				0.490
No	3,186 (95.19)	2,845 (95.28)	341 (94.46)	
Yes	161 (4.81)	141 (4.72)	20 (5.54)	
Arthritis				0.070
No	2,826 (84.43)	2,533 (84.83)	293 (81.16)	
Yes	521 (15.57)	453 (15.17)	68 (18.84)	
Asthma				0.002
No	3,243 (96.89)	2,903 (97.22)	340 (94.18)	
Yes	104 (3.11)	83 (2.78)	21 (5.82)	
Depression				0.680
No	1,893 (56.56)	1,696 (56.80)	197 (54.57)	
Yes	1,442 (43.08)	1,279 (42.83)	163 (45.15)	
Satisfaction				0.120
Satisfied	2,745 (82.01)	2,459 (82.35)	286 (79.22)	
Not satisfied	433 (12.94)	384 (12.86)	49 (13.57)	

Data are presented as number (proportion, %).

Missing data: depressive symptoms = 12, satisfaction = 169.

### Associated factors

**FIGURE 1:**
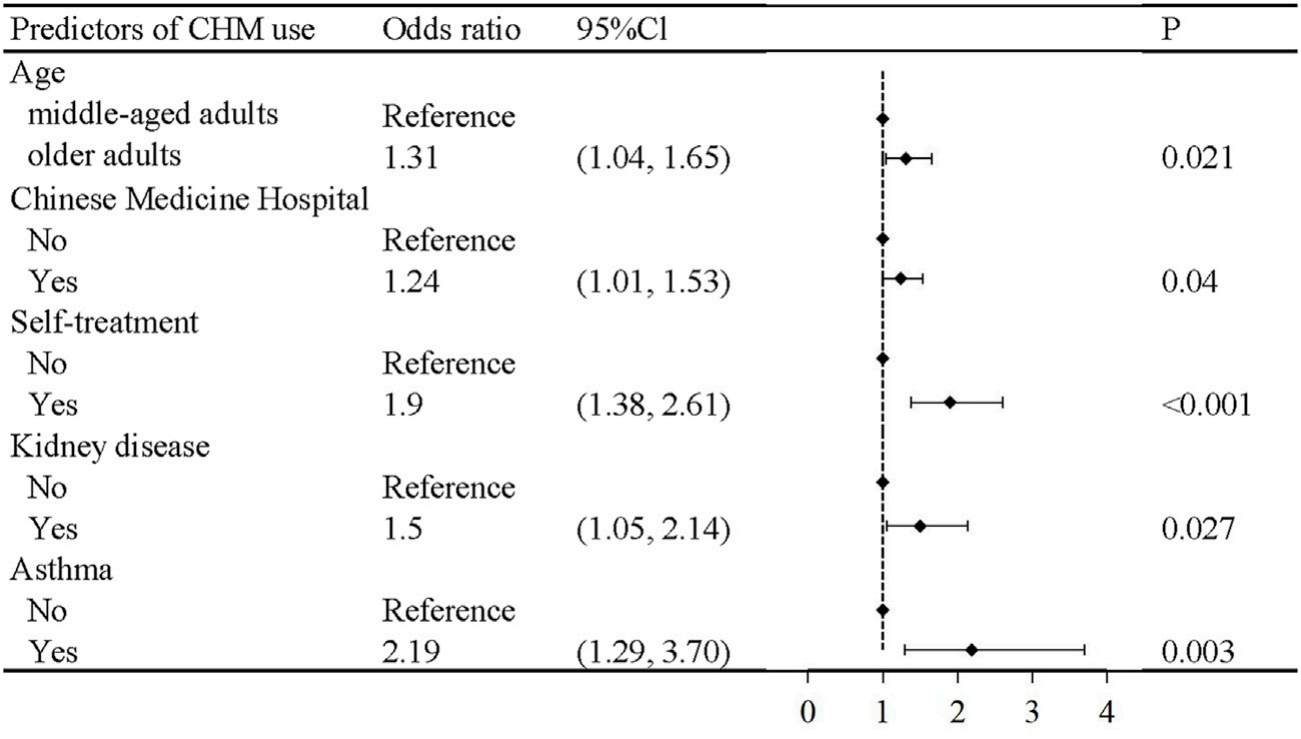
Logistic regression analysis for predictors of CHM use in Chinese middle-aged and older adults with diabetes.

The stepwise backward logistic regression model showed that diabetic patients who were older (OR = 1.31, 95% CI = 1.04, 1.65), visited Chinese medicine hospitals (OR = 1.24, 95% CI = 1.01, 1.53), and engaged in self-treatment (OR = 1.90, 95% CI = 1.38, 2.61) had higher likelihood of CHM use compared to those who were middle-aged, did not visit Chinese medicine hospitals, or engage in self-treatment. Further, participants with kidney disease (OR = 1.50, 95% Cl = 1.05, 2.14) and asthma (OR = 2.19, 95% Cl = 1.29, 3.70) were more likely to use CHM compared with those without these diseases (Figure 1).

Subgroup analyses were performed by age, gender, educational level, and residence region. The results of the subgroup analyses were consistent with the main results (Supplementary Table S2).

### Additive interaction between kidney disease and asthma

**FIGURE 2:**
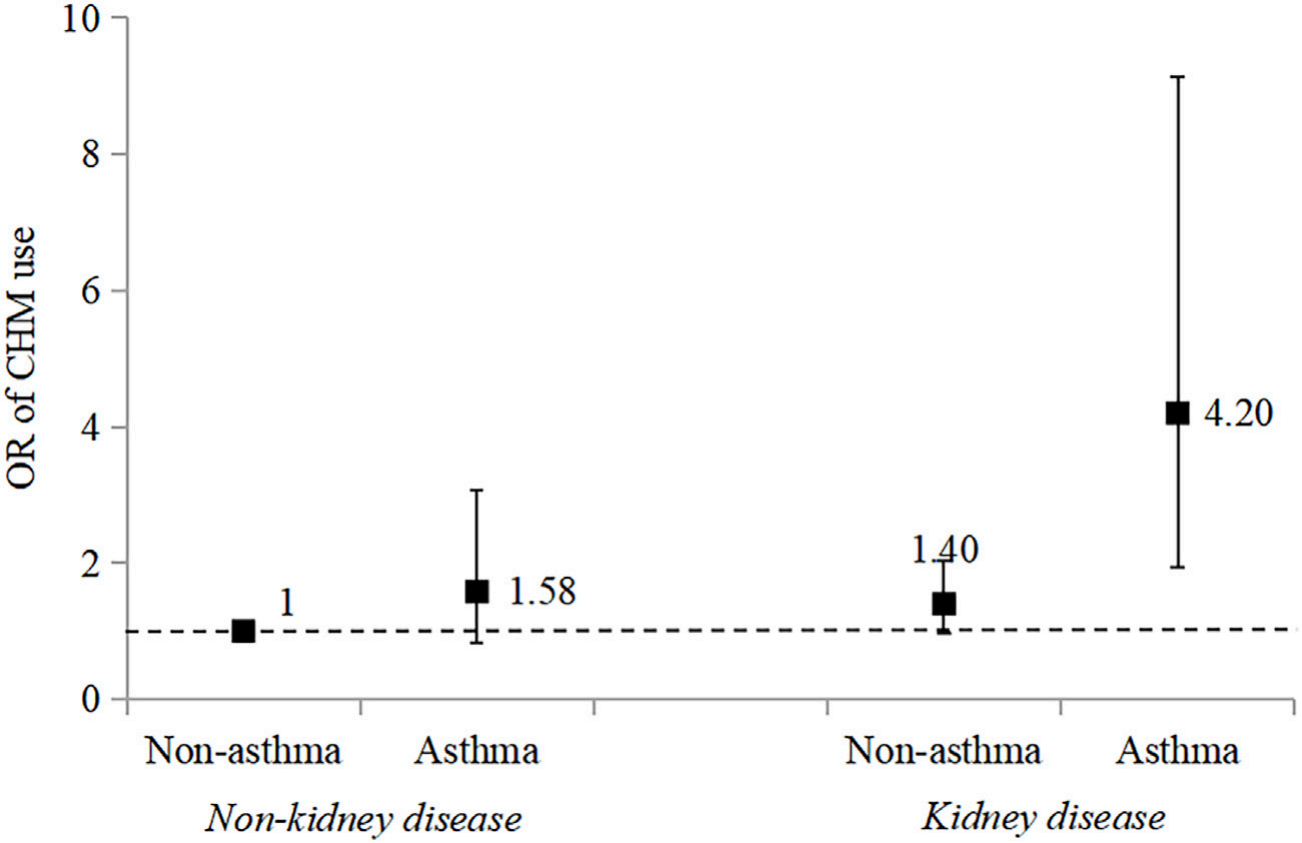
Combined effect of kidney disease and asthma on Chinese herbal medicine use in Chinese middle-aged and older adults with diabetes. Adjusted for received income, Chinese medicine hospital visits, self-treatment, general health status, diabetes, stroke, and kidney disease.

We performed additive interaction analyses using participants without kidney disease and asthma as a reference. The OR of CHM use among those with asthma was 4.20 (95% Cl = 1.93, 9.14) (Figure 2; Supplementary Table S1). There was a significant additive interaction between kidney disease and asthma on CHM use in diabetic patients (RERI: 2.22, 95% Cl = 0.65, 3.79; AP: 0.53, 95% Cl = 0.27, 0.79; S: 3.26, 95% Cl = 1.19, 8.98).

## Discussion

Diabetes is a common chronic disease globally and a leading cause of morbidity and mortality (Authors Anonymous, 2021). Western medical approaches to diabetes management are often associated with high risks of adverse drug reactions and are costly, making their use challenging in low-income countries (Liu et al., 2023). The use of CHM for diabetes treatment has gained recognition internationally (Pang et al., 2019; Liu et al., 2023; Shin et al., 2020; Ooi et al., 2012). The present study investigated the prevalence and associated factors of CHM use among middle-aged and elderly diabetic patients in China.

We found that the proportion of diabetic patients using CHM was relatively low at 10.8%. A similar study in Taiwan reported that 77.9% of diabetic patients used CHM, but only 13.9% used it specifically for diabetes (Huang et al., 2013), aligning with our findings. TCM categorizes diabetes as “Xiao Ke Syndrome.” This condition has been treated with TCM for over 2000 years in China, utilizing ancient formulas such as “Gegen Qinlian Decoction (GQD) (Tian et al., 2016)” and “Huanglian Jiedu decoction” (Hu et al., 2021). Modern scientific research has identified anti-inflammatory, antioxidant, lipid metabolism-regulating, and hypoglycemic effects in some herbal medicine components, which can effectively improve blood glucose levels and diabetes prognosis (Kim et al., 2020; Zare et al., 2019; Ceylan-Isik et al., 2008; Li et al., 2004).

In middle-aged and elderly diabetic patients, certain healthcare service variables significantly influence CHM use. Patients who engage in self-treatment or seek treatment at Chinese medicine hospitals are more likely to use CHM. In China, CHM is a traditional practice passed down through generations, making it widely accepted and used. Self-medicating patients with failure of Western treatments are often influenced by Chinese medicinal cultural and social factors (such as other people with diabetes, relatives, or friends), leading them to consider CHM as an alternative therapy (Burke et al., 2006; Choudhury et al., 2018). Furthermore, physicians at Chinese medicine hospitals prefer using CHM for treatment; therefore, patients seeking care at Chinese medicine hospitals are more likely to use CHM. However, a lower proportion of CHM users visited Chinese medicine hospitals, indicating a lack of consultation with Clinical Practitioners, which could lead to inadequate coordinated care for diabetes management.

Our results show that CHM users are more likely to have poor general health and to suffer from conditions such as stroke, kidney disease, or asthma compared to non-users. These findings suggest that patients with poor health status or multiple health problems are more likely to seek alternative treatments, similar to a previous study (Cauffield, 2000). CHM, an important type of alternative treatment, is effective for various chronic conditions, including diabetes, and has improved survival rates in diabetic patients (Lin et al., 2015). Furthermore, our interaction analysis confirmed that diabetic patients with both kidney disease and asthma were 4.2 times more likely to use CHM than those without these conditions, indicating that the presence of certain comorbidities is significantly associated with CHM use. Therefore, complementary therapies such as CHM are potentially useful strategies to address complex, multifaceted health conditions.

The strengths of this study are as follows. First, this is the first study to investigate the prevalence and associated factors of CHM use among middle-aged and elderly patients with diabetes in China using a nationally representative sample. Second, we have comprehensively analyzed the associated factors of CHM use among middle-aged and elderly patients with diabetes, including demographic characteristics, health status, and healthcare utilization. Third, our study demonstrated the combined effect of kidney disease and asthma on CHM use. These findings provide new insights and directions for future research on CHM use for diabetes treatment.

This cross-sectional study has several limitations. First, self-reported data were collected from patients, which may lead to recall bias. Second, the cross-sectional study design restricts our ability to establish causal relationships between associated variables and CHM use in patients with diabetes. Third, the cultural context and widespread acceptance of TCM in China may influence both the prevalence and patterns of CHM use, making it difficult to extrapolate these results to other countries where CHM may not be integrated in mainstream healthcare. Fourth, certain advanced statistical methods, such as Bayesian shrinkage models (Bhattacharyya et al., 2022), may offer a more powerful approach for variable selection in regression analysis. We did not employ these methods in our study, which is a potential limitation. Fifth, although we conducted subgroup analyses using traditional methods, emerging approaches such as Bayesian Decision-Theoretic Methods (Mitra et al., 2020) could further improve the subgroup analysis.

In light of these limitations, we suggest several directions for future research. First, as previous studies have indicated (Matthews et al., 2022; Bhattacharyya and Rai, 2019), clinical trials should be conducted to assess the safety and effectiveness of CHM use. Second, applying advanced methods such as Bayesian shrinkage models (Bhattacharyya et al., 2022), Bayesian subgroup analysis methods (Mitra et al., 2020), and prediction models could enhance the statistical analysis of CHM use and its associated factors. Third, interventions targeting the identified influencing factors can improve the utilization rate of CHM.

In summary, our findings indicate a relatively low utilization rate of CHM for diabetes treatment among middle-aged and elderly patients. CHM use was associated with specific comorbid conditions, preference for self-treatment, and visit to Chinese medicine hospitals. Our findings emphasize the role of specific health conditions and healthcare behaviors associated with CHM usage among diabetic patients.

## Data Availability

Publicly available datasets were analyzed in this study. This data can be found here: CHARLS, http://charls.pku.edu.cn/.
