# Supplementary material for: Prevalence and factors associated with Chinese herbal medicine use among middle-aged and older Chinese adults with diabetes mellitus
Source: Front Pharmacol. 2025 May 22;16:1482228. doi: 10.3389/fphar.2025.1482228 (PMC12137263; doi:10.3389/fphar.2025.1482228)
Supplement: Supplementary file 1 [file Table1.docx]

**Supplementary Table 1.** Combined effect of kidney disease and asthma on Chinese herbal medicine use in Chinese middle-aged and older adults with diabetes.

| Joint exposure | | N | Chinese herbal medicine use | | |
| --- | --- | --- | --- | --- | --- |
| Kidney disease | Asthma |  | n | OR ^a^ | P |
| No | No | 2986 | 303 | Reference |  |
| Yes | No | 257 | 37 | 1.40 (0.96, 2.03) | 0.077 |
| No | Yes | 72 | 11 | 1.58 (0.82, 3.06) | 0.170 |
| Yes | Yes | 32 | 10 | 4.20 (1.93, 9.14) | < 0.001 |

^a^ Adjusted for received income, Chinese medicine hospital visits, self-treatment, general health status, diabetes, stroke, and kidney disease.

**Supplementary Table 2.** Subgroup analysis of significant predictors of CHM use among Chinese middle-aged and older adults with diabetes using Logistic regression.

| Subgroup | Predictors of CHM use | Odds ratio | 95%Cl | P |
| --- | --- | --- | --- | --- |
| Middle-aged adults | Chinese Medicine hospital visits |  |  |  |
|  | No | Reference |  |  |
|  | Yes | 1.53 | (1.17, 1.99) | 0.002 |
|  | Self-treatment |  |  |  |
|  | No | Reference |  |  |
|  | Yes | 2.01 | (1.25, 3.25) | 0.004 |
|  | Kidney disease |  |  |  |
|  | No | Reference |  |  |
|  | Yes | 1.88 | (1.12, 3.16) | 0.017 |
| Older adults | Self-treatment |  |  |  |
|  | No | Reference |  |  |
|  | Yes | 1.80 | (1.17, 2.76) | 0.007 |
|  | Asthma |  |  |  |
|  | No | Reference |  |  |
|  | Yes | 2.14 | (1.27, 4.89) | 0.008 |
| Male | Self-treatment |  |  |  |
|  | No | Reference |  |  |
|  | Yes | 1.84 | (1.18, 2.86) | 0.007 |
|  | Asthma |  |  |  |
|  | No | Reference |  |  |
|  | Yes | 3.87 | (1.89, 7.90) | <0.001 |
| Female | Chinese Medicine hospital visits |  |  |  |
|  | No | Reference |  |  |
|  | Yes | 1.33 | (1.00, 1.76) | 0.049 |
|  | Self-treatment |  |  |  |
|  | No | Reference |  |  |
|  | Yes | 1.90 | (1.19, 3.03) | 0.008 |
|  | Kidney disease |  |  |  |
|  | No | Reference |  |  |
|  | Yes | 1.74 | (1.07, 2.84) | 0.025 |
| Illiterate, Primary school and below | Age |  |  |  |
|  | Middle-aged adults | Reference |  |  |
|  | Older adults | 1.39 | (1.01, 1.92) | 0.045 |
|  | Self-treatment |  |  |  |
|  | No | Reference |  |  |
|  | Yes | 1.90 | (1.38, 2.61) | <0.001 |
| Middle school and above | Self-treatment |  |  |  |
|  | No | Reference |  |  |
|  | Yes | 1.64 | (1.06, 2.53) | 0.027 |
|  | Asthma |  |  |  |
|  | No | Reference |  |  |
|  | Yes | 3.29 | (1.55, 7.01) | 0.002 |
| Urban | Age |  |  |  |
|  | Middle-aged adults | Reference |  |  |
|  | Older adults | 1.78 | (1.10, 2.86) | 0.018 |
|  | Self-treatment |  |  |  |
|  | No | Reference |  |  |
|  | Yes | 2.46 | (1.24, 4.89) | 0.010 |
|  | Stroke |  |  |  |
|  | No | Reference |  |  |
|  | Yes | 2.60 | (1.39, 4.87) | 0.003 |
| Village | Chinese Medicine hospital visits |  |  |  |
|  | No | Reference |  |  |
|  | Yes | 1.43 | (1.12, 1.83) | 0.004 |
|  | Self-treatment |  |  |  |
|  | No | Reference |  |  |
|  | Yes | 1.69 | (1.18, 2.43) | 0.004 |
|  | Asthma |  |  |  |
|  | No | Reference |  |  |
|  | Yes | 2.32 | (1.31, 4.11) | 0.004 |
